# Essential oil production from seeds of carrot (*Daucus carota* L.) grown on phytomanaged trace element-contaminated soils

**DOI:** 10.1007/s11356-026-37466-9

**Published:** 2026-02-07

**Authors:** Abderrahmane Hadini, Frédéric Laruelle, Natacha Facon, Dorothée Dewaele, Joël Fontaine, Anissa Lounès-Hadj Sahraoui

**Affiliations:** 1 Unité de Chimie Environnementale Et Interactions Sur Le Vivant (UCEIV - UR 4492), Universite du Littoral Côte d’Opale, 50 Rue Ferdinand Buisson, 62228 Calais Cedex, France; 2 Centre Commun de Mesures, Université du Littoral Côte d’Opale, 59140 Dunkirk, France

**Keywords:** *Daucus carota* L., Phytomanagement, Polluted soils, Trace elements (TE), Essential oil, Microbial biomass

## Abstract

The cultivation of *Daucus carota* L. (carrot)  on soils polluted with trace elements (TE) constitutes an innovative phytomanagement approach, combining significant ecological and economic benefits. This plant species exhibits a notable capacity to extract trace elements from the soil while generating valuable biomass, primarily intended for hydrodistillation to produce high-quality essential oils (EO). Carrots exhibit an enhanced capacity to accumulate cadmium in their leaf tissues, with a high bioconcentration factor (BCF = 1.71), as well as significant zinc accumulation. Furthermore, the cultivation of this biennial plant promotes an increase in soil microbial biomass, as assessed by phospholipid fatty acid (PLFA) content, specifically enriching populations of Gram− bacteria, Gram+ bacteria, and saprotrophic fungi, thereby contributing to the modulation of microbial dynamics and the alleviation of stress within soil microbial communities. The EO extracted from carrot seeds contain TE concentrations below the quantification limit compared to other commercially available EO. Chemical analysis of the EO reveals a composition similar to that of commercial oils, with the major components being carotol (27.53%), an oxygenated sesquiterpene, and sabinene (26.08%), a monoterpene hydrocarbon. These EO have been extensively evaluated for their biological properties, demonstrating significant antifungal activity against *Fusarium culmorum* (IC50 = 0.57 ± 0.11 mg/ml) and *Zymoseptoria tritici* (IC50 = 1.09 ± 0.12 mg/ml), as well as antigermination activity against *Blumeria graminis* spores (IC50 = 1.47 ± 0.15 mg/ml). Additionally, these EO exhibit notable herbicidal properties, particularly in inhibiting root elongation and germination of the monocots species *Lolium perenne* (ryegrass) and the dicots species *Lactuca sativa* (lettuce). These findings highlight the potential of carrot as a tool for phytoremediation and the production of high-value bioactive compounds.

**Graphical Abstract:**

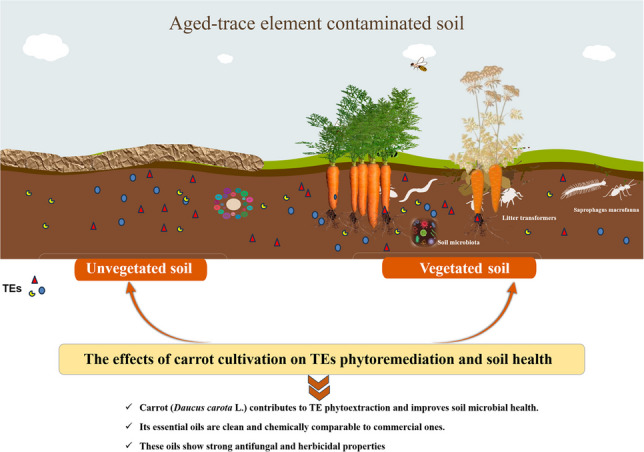

**Supplementary Information:**

The online version contains supplementary material available at 10.1007/s11356-026-37466-9.

## I. Introduction

Soil contamination has become an increasingly complex environmental challenge, driven by industrial, agricultural, and urban activities that introduce a diverse array of pollutants into ecosystems (Alloway, 2013; Gavrilescu 2022). These include organic contaminants, such as pesticides and petroleum derivatives, which compromise soil fertility and disrupt microbial communities, as well as emerging pollutants like pharmaceuticals, microplastics, and other anthropogenic compounds that threaten soil stability, food safety, and overall ecosystem health (Zhang and Wang 2020; Rillig et al. 2017). Trace elements (TEs) are also commonly detected in aged contaminated soils, originating from multiple sources including automotive traffic, industrial and mining activities, pesticide application, petrochemical spills, and disposal of pollutant-rich waste (Gavrilescu 2022; Zhang and Wang 2020). Their presence in soils adversely affects plant growth and development and disrupts the activity of key soil microorganisms, which play a central role in maintaining soil health and ecosystem functionality (Ferreira et al. 2022). Furthermore, the accumulation of TEs in crops destined for human or animal consumption can lead to food chain contamination, posing serious health risks. These considerations underscore the critical importance of implementing effective remediation strategies for polluted soils (Antoniadis et al. 2017; Mitra et al. 2022).

The use of physical or chemical processes to decontaminate heavily TE-polluted soils is an expensive procedure. It can often lead to ecosystem degradation and poses a potential risk of pollution transfer when the excavated soil is moved for off-site treatment (Cunningham and Berti 2020; Kumpiene et al. 2019). Consequently, current approaches to soil remediation increasingly favor environmentally friendly alternatives such as phytomanagement (Alkorta et al. 2004). In the context of TE pollution, this includes strategies like phytoextraction and phytostabilization (Cheraghi et al. 2011; Yang et al. 2016). While phytoextraction involves the uptake of TE into the above-ground biomass of plants, phytostabilization focuses on limiting the mobility of these elements within the soil without removing them (Awa and Hadibarata 2020).

Moreover, the selection of appropriate plant species is crucial in a phytomanagement approach. The chosen plants should be able to tolerate high concentrations of TE and should produce significant biomass to consider its valorization (Kafle et al. 2022). Indeed, the interest of the phytomanagement approach consists in the combination of the phytoremediation with the valorization of the biomass produced on polluted soils as well as the ecological rehabilitation of the polluted soil (Edgar et al. 2021). In this regard, the cultivation of aromatic plants, in particular sage, angelica, and coriander, destined for essential oil (EO) production has been presented as an innovative and economically viable alternative for reclaiming TE polluted areas (Langrand et al. 2025; Perlein et al. 2021; Raveau et al. 2022). The global demand for EO is currently increasing significantly (Assadpour et al. 2023; Sundar and Parikh 2023). These high-value-added products are widely used as aromatic agents in various non-food industries, such as perfumery, cosmetics, and medicine, as well as potential crop-protection products, which could provide economic benefits with the produced biomass (Liñán-Atero et al. 2024; Rybczyńska-Tkaczyk et al. 2023).

Carrots (*Daucus carota*), a diploid and biennial vegetable from the Apiaceae family originating from Afghanistan and nowadays cultivated in nearly every country in the world, are well known for their nutritional qualities and are also a strategic plant resource for the extraction of high value-added molecules. They are highly valued for their nutritional benefits, particularly as a major source of β-carotene and vitamins (Koley et al. 2014; Önder et al. 2024). This root vegetable, grown worldwide, has a particularly rich biochemical composition that is attracting growing interest from the pharmaceutical, cosmetics and nutraceutical sectors (Dehghani et al. 2025). Indeed, carrots are a rich source of high value-added molecules, particularly carotenoids, which have antioxidant and provitamin properties. They also contain bioactive polyacetylenes with antimicrobial and potentially anti-cancer effects. Their fiber, vitamins, sugars and volatile compounds are valuable in the cosmetic and pharmaceutical fields (Pérez et al. 2023). They are known to be rich in secondary metabolites, EO and natural antioxidants (Angelini et al. 2022; Šeregelj et al. 2020). They produce high added-value EO rich in monoterpenes such as geranyl acetate and α-pinene, and demonstrate significant antifungal, antibacterial, and anti-inflammatory activities (Badalamenti et al. 2022; Valente et al. 2015). In addition, recently, a study, in a pot experiment, examined how carrot plants respond to soil polluted with cadmium (Cd), lead (Pb), and zinc (Zn) from historic mining. The results showed that Cd and Zn accumulated mainly in the leaves, while Pb remained in the roots, leading to oxidative stress, reduced biomass, and changes in photosynthetic and metabolic activity (Novák et al. 2023).

The current study aims to study the phytomanagement potential of *Daucus carota* L. and to evaluate the relevance of using carrot seeds for producing EO in the context of the phytomanagement of aged TE-polluted soil. Thus, the following questions have been addressed:

(1) What is the phytoremediation ability of carrot (i.e., TE phytoextraction or phytostabilisation)?; (2) What is the yield and the quality (regarding chemical composition, metallic and pesticide contaminations, antifungal and herbicidal activities) of carrot EO produced on TE-polluted soil?; (3) What is the impact of carrot cultivation on the microbial biomass in TE-polluted soil?

## II. Materials and methods

### Experimental site

The study was carried out on a 1000 m^2^ TE-polluted site, located in the north of France at Evin-Malmaison (50° 25′ 55.5″ N, 3° 02′ 25.5″ E). The site is an agricultural field located 600 m from the former Metaleurop Nord smelter, which had been one of the main lead plants in Europe for decades (100 years), releasing large quantities of dust that heavily polluted the surrounding soil (Labidi et al. 2015). The soil is mainly polluted by Cd, Pb, and Zn, with concentrations measured at 7.2 ppm, 394 ppm, and 443 ppm, respectively; these concentrations are respectively 17, 11, and sixfold higher than those reported in the regional background levels for agricultural soils (Sterckeman et al. 2007). This soil exhibited an alkaline pH of 7.86 and a silty clay loam texture, with 26% clay and 53% silt. It contained relatively high levels of organic matter (OM) (29 g kg⁻^1^) and total organic carbon (17 g kg⁻^1^). The cation exchange capacity (CEC) was high (183 cmol⁺ kg⁻^1^), reflecting strong nutrient and metal retention capacity (Perlein et al. 2021). Despite historical TE-pollution, the studied soil sustains a biologically active and diverse microbial ecosystem, as previously demonstrated in our earlier studies (Raveau et al. 2020; 2021). The bacterial community is dominated by metal-tolerant phyla like Actinobacteria and Proteobacteria, with a significant portion consisting of plant growth-promoting rhizobacteria. Fungal diversity is notably high for a polluted site, particularly regarding arbuscular mycorrhizal fungi (AMF), with 69 identified species suggesting strong native adaptation.

### Experimental setup

*Daucus carota* L. (cultivar Nantaise, the seeds were kindly provided by Ferrant PHE) was sown in September 2022 and cultivated over an area of 800 m^2^ for 1 year until seed production. The experimental design also included a 200 m^2^ unvegetated area adjacent to the vegetated plot. In September 2023, plant and soil samples were collected according to a predetermined sampling plan. Nine (vegetated plot) and six (unvegetated plot) geo-referenced sampling points were established, evenly spaced to best represent the plots’ configuration (S1). For plant analysis, roots, stems, leaves, and seeds were collected and stored at 4 °C until use. For EO extraction, the umbels were harvested across the entire plot until sufficient biomass was obtained to perform the EO extraction. Soil samples of 500 mg were taken from a depth of 0–20 cm using an auger and stored at −20 °C until analysis.

### Soil and plant analysis

#### pH measurement

To measure the pH, 5 g of soil sieved to 2 mm was mixed with 25 mL of distilled water and stirred at room temperature for 2 h, following the NF ISO 0390 (2005) standard procedure. Once agitation was complete, the solution was allowed to rest for 1 h, after which the pH was measured (Fontaine et al., 2022a).

#### Determination of soil and plant total and extractable TE fractions

In conformity with the NF ISO 19730-2008-E standard, the bioavailable fraction of TE was evaluated. A selective extraction was carried out using 10 ± 0.01 g of 2 mm sieved soil suspended in 25 mL of 1 M ammonium nitrate (NH_4_NO_3_). The soil-extract mixture was agitated for 2 h, filtered using a 0.45 μm membrane, and the resulting leachates were acidified to pH 2. Analysis of both total and extractable TE concentrations was performed using Inductively Coupled Plasma Optical Emission Spectrometry (ICP-OES,) ICAP 6300 DUO, ThermoFisher Scientific, Loughborough, UK) and inductively coupled plasma mass spectrometry (ICP-MS, Varian 820 MS, Varian, USA), depending on TE concentrations.

The total TE concentrations in soil samples were determined following the NF-EN-13656 standard, using microwave digestion (Multiwave 3000 by Anton Paar, in St. Albans, UK). For this, 0.5 g of soil was digested at 180 °C for 20 min in a mixture consisting of 6 mL of hydrochloric acid (36%), 2 mL of nitric acid (67%), and 2 mL of hydrofluoric acid (48%). After digestion, the mixture was filtered using 0.45 mm Whatman paper, and the resulting extract was stored until analysis.

The determination of the total fraction of TE in the plant involved analyzing all its parts (roots, stems, leaves, and seeds) as well as the residues remaining after the extraction of essential oils, using 0.2 g of dry matter. Subsequently, digestion at 180 °C for 20 min in a mixture of 10 mL of nitric acid (67%) and 3 mL of ultra-pure water using a microwave digester was performed. Two standard reference materials, BCR-679 (white cabbage) and ERM-CC141 (loam soil), from Sigma Aldrich, St. Louis, MO, were utilized for all analytical quality control.

Calculation of the Bioconcentration Factor (BCF) for Cd, Zn, Pb, and Cu, from soils to vegetables, was carried out using the following formula:

$$\begin{aligned}BCF\:&=\:Metal\;concentration\;in\;plants\\& /Total\;metal\;concentration\;in\;soil.The\;ratio\;o\;BCF\:\\&>\:1\;indicates\;a\;higher\;accumulation\;of\;metals\;in\;plant\;parts.\end{aligned}$$

The translocation factor (TF) was also calculated and served as an indicator of the efficiency with which plants transfer metals from root tissues to shoots. It was determined as the ratio of metal concentration in the shoot to that in the root: TF = [Metal] shoot **/** [Metal] root.

The percentage of mobility of TE is also calculated as follows:

$$\%\;mobility\:=\:extractable\;TE\;concentration\;in\;soil/total\;TE\;concentration\;in\;soil\;\ast100$$

### Analysis of soil phospholipid fatty acids (PLFA)

The extraction of Phospholipid Fatty Acid (PLFA) was based on the methodology of Blight and Dyer first proposed in 1959 with some modifications (White et al. 1979). A quantity of 3 g of lyophilized soil samples was extracted with a mixture of chloroform-methanol-citrate buffer (1/2/0.8, v/v/v). Lipids were separated into three fractions: (i) neutral lipids, (ii) glycolipids, and (iii) polar lipids, including phospholipids. For this purpose, silica-containing solid-phase extraction columns (Interchim, France) were used, with successive elutions employing, respectively, chloroform, acetone, and methanol (in a ratio of 1/2/1, v/v/v). The PLFA and Neutral Lipid Fatty Acids (NLFA) were then converted to methyl esters using a solution of 0.2 M KOH in methanol (Zelles 1999). These methyl esters were analyzed using a gas chromatograph-mass spectrometer (GC-MS) (QP 2010 Ultra, Shimadzu, Kyoto, Japan) equipped with a single quadrupole mass detector and a flame ionization detector (FID). The samples were injected in split mode (ratio 80/1) onto a fast ZB-1MS capillary column (Phenomenex, Torrance, California, USA) made of 100% dimethylpolysiloxane, with dimensions of 10 m in length, 0.1 mm in internal diameter, and 0.1 µm in phase thickness, using helium as the carrier gas at a constant linear velocity of 40 cm/s. The injector temperature was maintained at 280 °C, while the detector temperatures were 330 °C for the flame ionization detector and 280 °C for the ion source. The GC temperature program was started at 175 °C, increased at a rate of 25 °C/min to reach a final temperature of 275 °C over a period of 0.5 min. Ionization was performed by electron impact at 70 eV, with a swept mass range of 50–400 m/z. Single-impact monitoring mode was used simultaneously (Fontaine et al., 2022a). To quantify fatty acid methyl esters, nonadecanoic acid methyl ester (20 µg/µl) was used as an internal standard (supplied by Sigma Aldrich). The fatty acids were identified by comparing their relative retention times with those of commercial standards (47080-U Bacterial Acid Methyl Ester (BAME) Mix, Sigma Aldrich) and by comparing the spectra obtained with the spectra of these commercial standards and those reported in the literature (NIST Standard Reference Database). The soil microbial biomass was evaluated using specific PLFA as biomarkers, whose quantities reflect the total living microbial cell biomass present in the soil. The sums of the masses of i15:0, a15:0, i16:0, i17:0, a17:0, and cy17:0, C18:1ω7, cy19:0 were calculated to quantify the biomass of Gram+ and Gram− bacteria, respectively (Frostegård et al. 1991; Larsen et al. 1998). For AMF and ectomycorrhizal and saprotrophic fungi, a single biomarker is studied to assess their total living cell biomass in the soil, which is C16:1ω5 and C18:2ω6,9, respectively (Joergensen 2022).

In the soil, the PLFA C16:1ω5 can originate from bacterial or fungal (AMF) sources (Olsson and Wilhelmsson 2000). Therefore, it is necessary to calculate the following ratio to determine the origin of the C16:1ω5 marker found in the sample: NLFA (C16:1⍵5) / PLFA (C16:1⍵5).

When the ratio is greater than 1, the marker originates from fungi, while when it is less than 1, the marker originates from bacteria. Total microbial biomass was estimated from the sum of PLFA content, while the ratio of cyclopropyl PLFA to their monoenoic precursors (cy17:0+cy19:0)/(16:1ω7+18:1ω7) served as an indicator of physiological or nutritional stress within bacterial communities (Carrasco et al. 2010). Indeed, this ratio compares cyclopropyl fatty acids (cy17:0 and cy19:0), which increase under stress to stabilize bacterial membranes, to common mono-unsaturated fatty acids (16:1ω7 and 18:1ω7), that dominate in healthy, actively growing bacteria.

###  Essential oil extraction

The EO extraction from carrot seeds, utilizing 200 g for each of the 11 total extractions, was conducted through hydrodistillation in 4 L of distilled water. Each extraction lasted 4 h using a Clevenger-type apparatus (Khajeh et al. 2004). The EO extracted from each distillation was collected and then stored in sealed dark bottles at a temperature of 4 °C prior to GC-MS analysis. The essential oil yield was calculated using the following formula: Yield (%) = (weight of extracted essential oil (g) / weight of dried plant material (g)) × 100.

####  Essential oil analysis

The EO composition was analyzed using GC-MS (QP 2010 Ultra, Shimadzu, Kyoto, Japan) equipped with a single quadrupole mass detector and a flame ionization detector (FID). Helium was used as the carrier gas to maintain a constant flow rate (60 cm/s) in the system (Raveau et al. 2022). The oil was previously diluted in hexane (1:200 v/v) before being subjected to analysis. Then, an injection of 0.2 µL of the diluted carrot seed oil was made in split mode (1:15 v/v at a temperature of 220 °C) into a ZB-5MS capillary column, supplied by Phenomenex (Le Pecq, France), consisting of 5% phenylarylene and 95% dimethylpolysiloxane, with dimensions of 10 m in length, 0.10 mm in inner diameter, and a phase thickness of 0.10 µm. The temperature was programmed to start at a temperature of 60 °C (30 s) and then increase linearly at 25 °C min^−1^ to reach 315 °C, finally held for 1 min. Mass spectra were acquired using an ionization energy of 70 eV with an interface temperature also set at 280 °C. The mass range scanned was from 35to 600 m/z. To identify the components of the essential oil, Kovats indices were determined by comparing the retention times and the results of co-injecting a series of n-alkanes of a commercial calibration standard with chain lengths between 8 and 40 carbon atoms (C8-C40 Alkanes Calibration STD - Supelco 40147-U) with established data in the literature and The Pherobase database (https://www.pherobase.com/database/compound/compounds-index.php). The EO constituent percentages were deduced from the peak areas on the GC-FID chromatogram.

####  Essential oil quality evaluation

The quality assessment of EO extracted from carrot seeds grown in soils polluted by TE was conducted in comparison with commercial EO from carrots grown in non-polluted soils (Ferrant PHE Sarl https://www.ferrantphe.com/production-de-concentres/Rodelinghem, Haut de France). The comparison was based on determining the concentrations of TE in each oil and analyzing their chemical compositions, considering that both oils originate from the same carrot variety.

The analysis of TE in distilled EO was carried out by taking 5 mL in volume, and subjecting them to analysis by ICP-MS (Agilent 7500) after digestion (Raveau et al. 2021). The digestion process involved a mixture of 0.5 g of EO, 10 mL of HNO_3_, and 3 mL of pure water, heated in a microwave oven (Multiwave 3000, Anton Paar, St. Albans, UK) at 180 °C for 20 min.

###  Essential oil antifungal activity

Antifungal activity of carrot seed EO was evaluated *in vitro* via direct contact against two wheat phytopathogenic fungi, *Fusarium culmorum* in a Petri dish (El-Alam et al. 2020; Znini et al. 2013), and *Zymoseptoria tritici* in a liquid medium.

To carry out the antifungal test, 0.5 cm mycelial discs of *F. culmorum* were taken from a 6–7 days young fungal colony with 3 replicates and placed in the center of a 55 mm Petri dish containing sterile culture medium, which is potato dextrose agar (PDA, 40 g/L). Then, a series of concentrations of carrot seeds EO (0.004% to 1%) was prepared with three replicates, followed by an incubation at 20 °C for 4 days. At the end of the incubation period, the diameter of fungal growth was measured, presenting the inhibition rate calculated by using the following formula:

$$IR(\%)\:=\:(X0-X1)/X0\:\times\:100,$$

where X_0_ represents the average diameter of the fungal colony in the control and X_1_ indicates the average diameter of the fungal colony. The EO was solubilized using DMSO 1% (v/v) and compared with a positive control, i.e., a product that has shown its antifungal properties and is being used as a fungicide, namely Aviator XPro, with the same proportions of concentrations.

A graphical analysis method was used to determine the semi-maximal inhibitory concentration (IC50).

*In vitro* assays also investigated the antifungal efficacy of carrot seed EO against *Z. tritici*. Spores were harvested and placed in a glucose-peptone solution, then inoculated into microplates. These plates were incubated in darkness at a controlled temperature of 20 °C for 6 to 7 days with agitation (Clerck et al. 2020). Fungal growth was measured using a spectrometer at 620 nm. Each concentration (0.004% to 1%) of EO was tested in eight replicate wells. Additionally, 1% DMSO was used to solubilize the EO. Finally, the results were compared to those of a commercial fungicide product, Aviator XPro, in the same concentration range. The net optical density was calculated using the following formula:

$$Netoptical\;density\:=\:average\;optic\;density\;(OD)with\;spores\:-\:average(OD)\;of\;control\;without\;fungus$$

The IC50 was determined by generating interpolation graphs.

### Inhibition of *Blumeria graminis* spore germination

The inhibitory effect of the carrot seed EO on germination was evaluated through direct contact with *B. graminis* conidia, the pathogen responsible for wheat powdery mildew, under in vitro conditions. A range of concentrations of essential oils of carrot seeds was prepared (0.004% to 1%) with three repetitions, and added to a sterile medium of Agar (15 g/L – DMSO 1%). The *B. graminis* spores were directly dispersed onto the plates and observed using an optical microscope (Nikon Eslipse E600) after 48 h. The spores were classified as ungerminated or germinated, and their numbers were recorded ten times per Petri dish to calculate the average (Akachoud et al. 2022). Aviator XPro was used as a positive control to compare the effects of the oils. The IC50 was calculated by generating interpolation graphs.

### Evaluation of the herbicidal potential of  essential oil

The effects of carrot seed EO were evaluated on the seedling emergence and growth of two plant species, *Lolium perenne* L. (ryegrass) and *Lactuca sativa* L. (lettuce), respectively, a monocotyledonous and a dicotyledonous plant. These two species are commonly used in phytotoxicity tests and are listed in the Organization for Economic Co-operation and Development (OECD) guidelines (2003) for the assessment of chemicals (Judd et al. 2015; Paul et al. 2001).

EO were incorporated into an agar medium, cooled to 50 °C, and then poured into square Petri dishes measuring 12 cm on each side. Various concentrations of EO, ranging from 0.004% to 1%, were tested with a final volume of 60 mL per dish. DMSO was used to facilitate the dissolution of the essential oils. Two negative controls were set up for each species: one containing only agar in an aqueous solution in the same proportions as the tested samples, and another containing agar/DMSO at 1% in an aqueous solution. A positive control using an herbicide (Glyphosate) was also included. Once the medium solidified, lettuce seeds (12 per dish) or ryegrass seeds (10 per dish) were carefully placed on the surface of the agar, evenly spaced. The Petri dishes were placed vertically in growth chambers under controlled conditions for 8 days, a photoperiod of 16 h, a temperature of 20 °C in light and 12 °C in darkness, 70% humidity, and a light intensity of 4 LS (Yazdanbakhsh and Fisahn 2010).

After this incubation period, images of the dishes were taken using a “Winrhizo” scanner. The number of germinated seeds in each condition was counted, allowing the calculation of the IC50 value, determined by graphical interpolation.

### Statistical analysis

The statistical analysis was conducted using R software (version 4.3.2). Data normality was assessed using the Shapiro–Wilk test, as well as the Levene and Bartlett tests to verify the homogeneity of variances and homoscedasticity, respectively. A one-way analysis of variance (ANOVA) was performed to compare the means of different groups, primarily for the TE concentrations in soil and plant parts. This was followed by a student–Newman–Keuls (SNK) post-hoc test to identify specific differences between the groups at *p* < 0.05. The Student’s *t*-test was used to compare the means of microbial groups in addition to the other statistical analyses performed. The IC50 was determined by non-linear regression with 3 replicates. The values are expressed as Mean ± SD. In all these analyses, a *p*-value (*p* < 0.05) was considered statistically significant.

## III. Results and discussion

 The current study evaluates the extraction of high-value EO from carrots grown on aged TE-polluted soils as a non-food biomass valorization strategy within a phytomanagement framework.

### TE concentrations in soil and plant

The total TE concentrations (Cd, Pb, Zn, Cr, Cu, and Ni) were measured in the unvegetated and vegetated soils (Table 1), as well as in the various parts of the carrot plant after 12 months of cultivation (Table 2). As expected, the most abundant TE in soil was Zn and Pb. Carrot cultivation did not significantly alter the total concentrations of TE in the polluted soil (*p* < 0.05); however, it led to a significant increase in the bioavailable fractions of Cd and Zn. Specifically, the bioavailable Cd concentration rose from 0.05 μg/g dry soil (DS) in unvegetated soil to 0.08 μg/g DS, and Zn increased from 0.23 μg/g DS to 0.49 μg/g DS (*p* < 0.05). In contrast, no significant effect was observed on the bioavailable fractions of other TE (Pb, Cr, Cu, and Ni), which remained below the quantification limit (Table 1).

**Table 1:** Concentrations of total and bioavailable TE fractions (µg/g dry soil (DS)) in unvegetated and vegetated soils. Data are expressed as mean ± SD. Values not sharing a common letter indicate a significant difference at *p* < 0,05, (*t*-test)

Metals (μg/g DS)	Soil					
	**TE total fraction**		**TE Bioavailable fraction** **NH**_**4**_**NO**_**3**_**-extractable**		**Percent** (**%**) **of mobile TE calculated as extractable concentrations over total concentrations**	
	**Unvegetated soil**	**Vegetated soil**	**Unvegetated soil**	**Vegetated soil**	**Unvegetated soil**	**Vegetated soil**
**Cd**	6.66 ± 0.38^a^	6.18 ± 0.06^a^	0.05 ± 0.00^a^	0.08 ± 0.01^b^	0.75 ± 0.13^a^	1.30 ± 0.13^b^
**Pb**	394.67 ± 21.8^a^	382.61 ± 15.69^a^	< 0.05	< 0.05	0.01 ± 0.00^a^	0.01 ± 0.00^a^
**Zn**	427.71 ± 4.72^a^	397.54 ± 5.61^a^	0.23 ± 0.12^a^	0 0.49 ± 0.03^b^	0.05 ± 0.02^a^	0.12 ± 0.41^a^
**Cr**	80.64 ± 4.53^a^	80.04 ± 4.84^a^	< 0.025	< 0.025	0.03 ± 0.00^a^	0.03 ± 0.00^a^
**Cu**	26.44 ± 0.96^a^	24.98 ± 0.65^a^	< 0.025	< 0.025	0.10 ± 0.00^a^	0.10 ± 0.00^a^
**Ni**	22.23 ± 0.83^a^	22.25 ± 0.86^a^	< 0.025	< 0.025	0.11 ± 0.00^a^	0.11 ± 0.00^a^

**Table 2:** Concentrations of total TE (μg/g dry matter (DM)) in plant parts (roots, stems, leaves, seeds). Data are expressed as mean ± SD. Values not sharing a common letter indicate a significant difference (*p* < 0,05) based on SNK test

Metals (μg/g DM)	Carrot			
	**Roots**	**Stems**	**Leaves**	**Seeds**
**Cd**	5.66 ± 0.37^c^	4.64 ± 0.37^b^	10.63 ± 1.18^d^	1.96 ± 0.22^a^
**Pb**	6.10 ± 0.32^c^	2.10 ± 0.11^a^	20.17 ± 1.04^d^	3.54 ± 0.55^b^
**Zn**	30.86 ± 1.87^b^	17.30 ± 1.29^a^	135.04 ± 11.35^d^	70.01 ± 7.52^c^
**Cr**	0.85 ± 0.58^b^	0.58 ± 0.19^a^	2.63 ± 0.15^c^	2.94 ± 0.52^c^
**Cu**	5.87 ± 0.28^b^	1.74 ± 0.22^a^	16.28 ± 1.56^c^	18.58 ± 2.56^d^
**Ni**	< 1	< 1	3.23 ± 0.01^a^	3.12 ± 0.08^a^

The increase in the bioavailable fraction concentrations of Zn and Cd (*p* < 0.05) observed after carrot cultivation is consistent with the findings of a previous study conducted at the same site, which was previously cultivated with coriander (Fontaine et al., 2022a). This trend is commonly attributed in several studies to alterations in soil physicochemical properties, including pH, OM content, and CEC (Cui et al. 2020; Wang et al. 2004). In this study, however, soil pH remained stable (7.53 ± 0.11 for vegetated soil and 7.42 ± 0.05 for unvegetated soil), indicating that plant cultivation had no measurable effect on pH. Instead, the increase in the bioavailable fraction concentrations of Zn and Cd may relate to plant-specific mechanisms such as the release of root exudates, including chelating agents and low molecular weight organic acids (e.g., citric, oxalic, and malic acids), which enhance the bioavailability of TE by solubilizing nutrients and engaging in redox reactions (Chen et al. 2017; Javed et al. 2013). For instance, carrots are known to release metabolites into the rhizosphere to manage nutrient availability and metal stress (Chen et al. 2017; Luo et al. 2023), which can then be utilized as metabolites by microbes (Lee et al. 2019). Additionally, microbial communities in the rhizosphere can enhance the bioavailability and mobility of TE, potentially resulting in increased plant uptake (Andresen et al. 2015; Glick 2012). They achieve this by producing organic acids, siderophores, and other chelating compounds that solubilize metals and make them accessible to plants (Khan et al. 2019). Such microbial activity can amplify plant-mediated effects on metal extractability and accumulation, improving phytoextraction efficiency (Ma et al. 2016; Jing et al. 2020). These interactions likely explain the higher mobility of Cd observed in this study, consistent with its extractability, while other TE followed the mobility ranking Cd > Ni = Cu > Zn > Cr > Pb, with Pb being the least mobile (Table 1). Furthermore, the mobility of TE depends not only on their total soil concentration but also on their chemical forms, ranging from highly mobile water-soluble and exchangeable fractions to immobile residual fractions (He et al. 2005), emphasizing the complex interaction between soil properties, plant-root exudation, and microbial activity in regulating TE behavior.

**Fig. 1:**
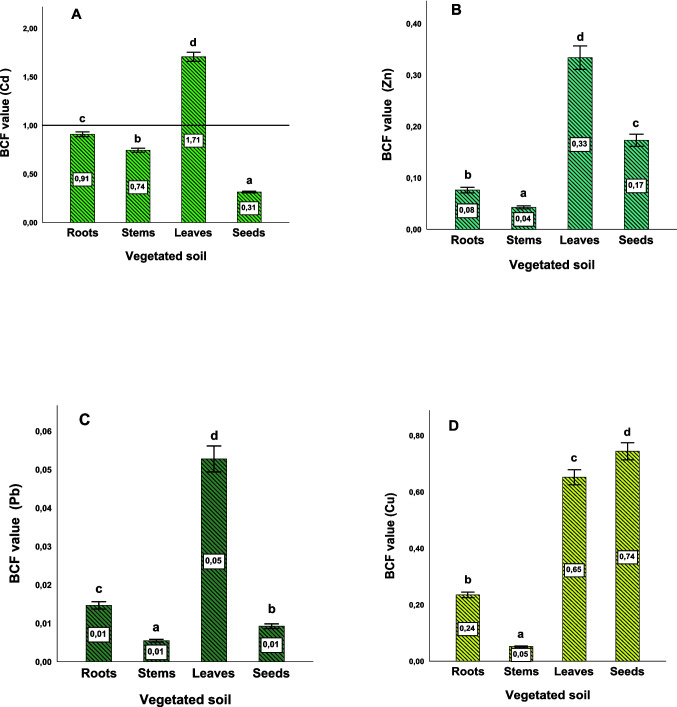
BCF values for Cd (**A**), Zn (**B**), Pb (**C**), and Cu (**D**) in roots, stems, leaves, and seeds. Data are expressed as mean ± SD. Values not sharing a common letter indicate a significant difference at *p* < 0.05 (SNK test)

The total concentrations of TE analyzed in the various parts of carrots (roots, stems, leaves, and seeds) showed a predominance of Zn accumulation in all parts of the plant (*p* < 0.05). The highest concentrations were observed in the leaves (135.04 ± 11.35 µg/g DM), followed by the seeds (70.01 ± 7.52 µg/g DM) for the same metal. Similarly, Cd is also accumulated in leaves at 10.63 ± 1.18 µg/g DM, which is higher than the levels recorded in the soil, while it accumulated in roots at 5.66 ± 0.37 µg/g DM (Table 2). The BCF calculated with the mean shoot Cd concentrations (BCF_tot_ = 1.71) in carrot leaves (Fig. 1) indicated an accumulator response for this element, which explains its mobility in the soil as reported in recent studies (Li et al. 2023; Ondrasek et al. 2020). For the other TE, an excluder response was indicated by the value of (BCF_tot_ < 1). However, the BCF value for Cd in our experimental conditions is higher than that found in carrots grown in other Cd-polluted soils (Yang et al. 2009), i.e., 0.62, due to the prolonged use of fertilizers containing Cd in the greenhouse in DaXing district, on the outskirts of Beijing, where vegetables have been cultivated for 15 years. Carrots have shown their capacity in the phytoremediation of soils polluted by TE, enhancing the absorption and translocation of these pollutants to the roots and aerial parts of the plants. Additionally, some of the TE concentrations decreased over time in the soil. Similarly, certain medicinal plants, such as *Lavandula dentata* L., *Thymus satureioides*, and *Teucrium polium* L., respond to polluted soils by accumulating TE (Boularbah et al. 2006). Recent studies have shown that aromatic plants such as coriander (*Coriandrum sativum* L.), angelica (*Angelica archangelica* L.), and clary sage (*Salvia sclarea* L.) can possess properties of extraction and accumulation of TE, especially Cd (Fontaine et al. 2022; Langrand et al. 2025; Raveau et al. 2021). Moreover, the efficiency of metal uptake depends on the plant species, the total metal concentration in the soil, the nature and bioavailability of the metal, as well as the soil physicochemical properties, which can also be altered by this interaction (Ait Elallem et al. 2021). Carrots have been used under different conditions than those of our research to test their tolerance to TE and they have been identified as accumulators, particularly of Cd and Zn, with significant accumulation observed in the roots, stems, and leaves (Sharma and Agrawal 2006), which aligns with the findings of the present study regarding Cd accumulation.

**Fig. 2:**
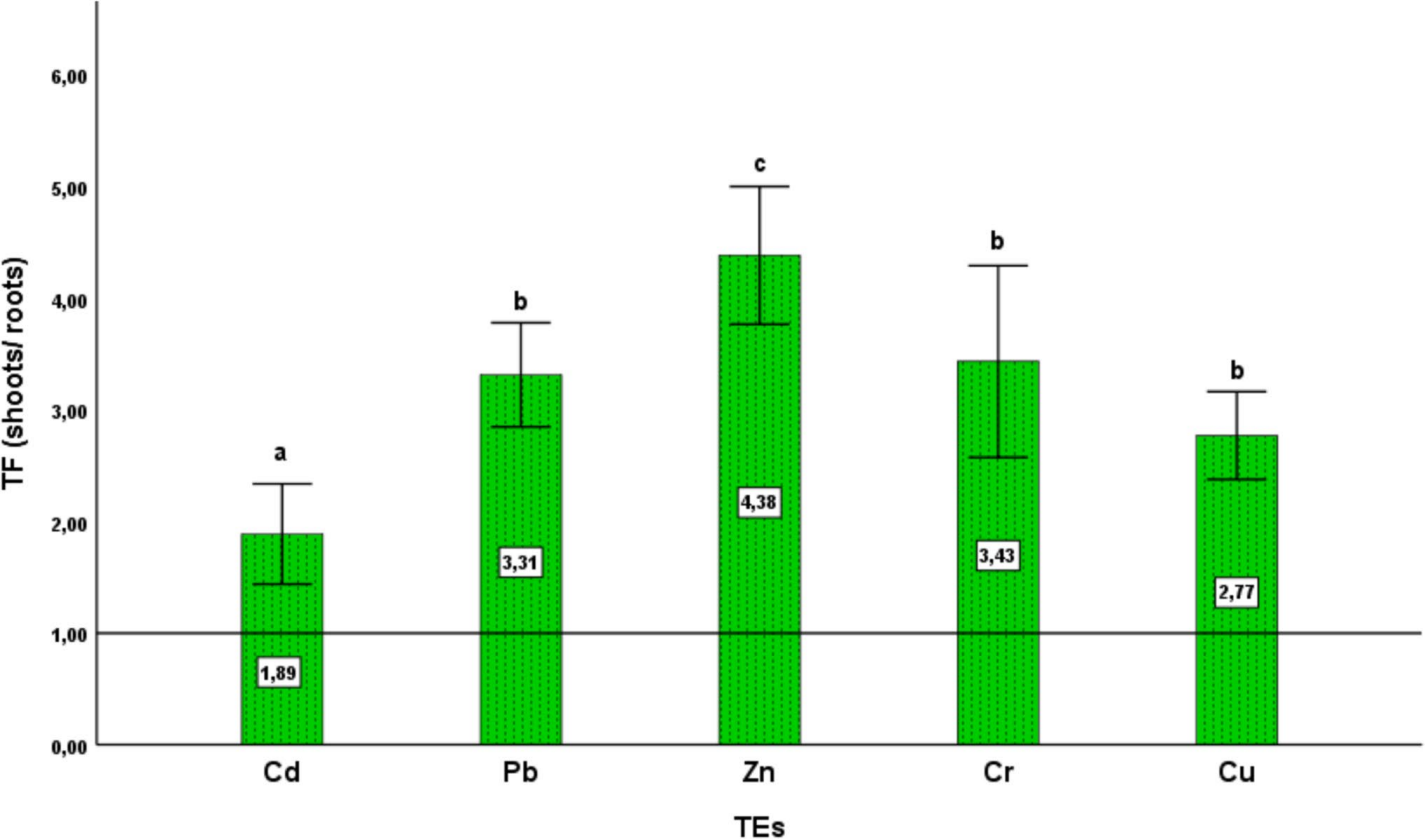
The translocation factor (TF) of TE in carrots after 1 year of cultivation. Results are presented as mean ± SD. Different lowercase letters above the columns indicate significant differences (*p* < 0.05; SNK test)

On the other hand, the TF of TE to the aerial parts of the carrot plant, calculated as the ratio between concentrations in leaves and roots, revealed interesting results, with TF > 1 for all the analyzed metals except for Ni, which showed concentrations below the quantification limit. Zinc exhibited the highest TF value, estimated at 4.38, indicating a strong capacity for translocation to the shoots. Similarly, Cr and Cu showed high TF values of 3.55 and 3.54, respectively (Fig. 2). In this context, the TF, especially when greater than 1, indicates effective translocation of TE to the aerial parts, which is essential for phytoextraction, whereas a TF < 1 reflects metal retention in the roots, which is more suitable for phytostabilization (Bini et al. 2012). In the present study, carrots showed a high Zn translocation capacity, which makes them suitable candidates for the extraction of this element from polluted soils. Moreover, they mainly transport Zn easily from roots to leaves, as it is an essential micronutrient involved in various metabolic functions (Drozdova et al. 2019). However, the accumulation of TE in the roots was lower, especially compared to the leaves and seeds. This accumulation is sometimes linearly dependent on the concentration of TE in the soil, as observed in our study for Zn. A similar trend was also reported in several studies (Malizia et al. 2012). Moreover, the response to TE accumulation can vary depending on the species, based on their physiology and their capacity for absorption and translocation (Nissim et al. 2018).

### Effect of carrot vegetation on microbial biomass in aged TE-polluted soil

Soil microbial communities are integral to ecosystem functioning and are particularly sensitive to environmental disturbances such as TE-pollution. Gaining insight into how plant cultivation affects these communities is crucial for designing effective phytomanagement approaches. Carrot cultivation, in particular, can support the restoration of soil functions impaired by metal pollution. Indeed, microbial communities are key players in numerous soil processes, including organic matter formation and decomposition, respiration, and nutrient cycling, all of which are vital for maintaining essential ecosystem services. In the present study, the total microbial biomass is significantly greater in soils where carrots are cultivated compared to unvegetated soils (Table 3). The biomass of the different microbial groups, including Gram− bacteria, Gram+ bacteria, and saprotrophic fungi, except AMF, increased significantly (*p* < 0,05) with carrot vegetation**.** An increase in the biomass of Gram− and Gram + bacteria was observed, which respectively increased from 1.88 ± 0.16 µg/g DS in the unvegetated soil to 2.64 ± 0.58 µg/g DS in the vegetated soil with carrot, and from 4.68 ± 0.55 µg/g DS in the unvegetated soil to 5.75 ± 0.19 µg/g DS in the vegetated soil. Although to a lesser extent than bacteria, saprotrophic fungi also show a significant increase in their biomass in vegetated soils from 0.35 ± 0.08 µg/g DS to 0.57 ± 0.11 µg/g DS. In contrast, the biomass of AMF did not differ significantly between vegetated and unvegetated soils. The difference observed between microbial groups could be attributed to their specific physiological characteristics and growth patterns, while fungi are not strongly influenced by nutrient availability, which likely explains their less pronounced response to vegetation restoration (Vos et al. 2013). Although TE can negatively affect microbial communities due to their toxic properties, the extent of this impact depends on several factors, such as the concentration and bioavailability of the metals, soil characteristics, and the resilience or adaptability of specific microbial groups. Indeed, exposure to high levels of TE can disrupt microbial diversity, reduce biomass, and impair essential soil functions such as nutrient cycling and organic matter decomposition (Xu et al. 2019). In our study, the cyclopropyl-to-cyclopropyl precursor ratio (a PLFA-based indicator of physiological stress in microbial communities, Choudhary et al. 2021) was lower in soils planted with carrots, highlighting the role of vegetation in modulating microbial dynamics and alleviating stress, as widely reported in the literature. Numerous studies have also shown that vegetation can enhance microbial biomass as well as the enzymatic activity of microbes in soil polluted by TE (Fontaine et al. 2022; Yu et al. 2016). Plants are characterized by the secretion of specific root exudates and rhizodeposits, shaping microbial community structures in the rhizosphere. Aromatic plant species also release secondary metabolites with antimicrobial properties into the soil, thus potentially playing a significant role in shaping bacterial communities (Misra et al. 2019; Raveau et al. 2020). In other studies investigating bacterial diversity and biochemical properties in the rhizospheric soils of cumin and coriander, metabarcoding techniques were used to demonstrate a direct correlation between microbial profiles and factors such as crop root exudates, soil organic carbon, and nitrogen levels.

**Table 3:** Biomass (µg/g dry soil) of specific microbial groups (Gram+ bacteria, Gram− bacteria, saprotrophic fungi, and arbuscular mycorrhizal fungi (AMF)), total microbial biomass, and stress indicator in the vegetated and unvegetated soils (*n* = 3). Data are expressed as mean ± standard deviation. Values not sharing a common letter indicate a significant difference (*p* < 0,05) according to *t*-test

	**Gram+ bacteria PLFA (µg/g)**	**Gram− bacteria PLFA (µg/g)**	**Saprotrophic fungi PLFA (µg/g)**	**AMF** **PLFA (µg/g)**	**Total microbial ∑ PLFA (µg/g)**	**Stress indicator**
Unvegetated soil	1.88 ± 0.16^**a**^	4.68 ± 0.55^**a**^	0.35 ± 0.08^a^	0.49 ± 0.03^**a**^	7.43 ± 0.57^**a**^	1.63 ± 0.12^**a**^
Vegetated soil	2.64 ± 0.58^**b**^	5.75 ± 0.19^**b**^	0.57 ± 0.11^**b**^	0.52 ± 0.09^**a**^	9.49 ± 0.96^**b**^	1.44 ± 0.16^**b**^

### Effect of TE-pollution on the yield and composition of essential oil distillated from *Daucus carota* L. seeds

Hydrodistillation of mature carrot seeds cultivated in TE-polluted soils yielded 5.91 mL/kg of EO (using 200 g per extraction). The extraction yield of 0.75 ± 0.10% falls within the range of 0.5% to 1.8% (v/w) reported in other studies using the same extraction method (Staniszewska et al. 2005; Verma et al. 2014). It is well established that extraction yields can vary depending on several factors such as the extraction method, plant variety, and the cultivation conditions (Alves-Silva et al., 2016).

Regarding the chemical composition, over 30 compounds were identified in the EO from carrot seeds grown in TE-polluted soils (Table 4). Analyses revealed distinct chemical profiles between the two oil types. The EO from TE-polluted soils was dominated by monoterpene hydrocarbons (56.01%) and oxygenated sesquiterpenes (31.06%). In contrast, the commercial EO from non-polluted soils (Ferrant PHE, France) was predominantly composed of oxygenated sesquiterpenes (41.32%) and sesquiterpene hydrocarbons (28.06%). Specifically, the major component in the EO from polluted soils was carotol (27.35%), followed by sabinene (26.08%), α-pinene (9.77%), D-limonene (5.92%), and β-myrcene (5.9%), as well as β-pinene (3.99%) (S2). Although the commercial carrot EO contains almost the same compounds, their proportions differ. Carotol remains the major component (38.23%), but secondary compounds appear in lower concentrations compared to the polluted soil EO, notably sabinene (8.29%) and α-pinene (5.89%), while others like β-caryophyllene (7.33%) and β-selinene (4.65%) are more prominent.

**Table 4:** Chemical composition (%) of essential oils from *Daucus carota* L. seeds cultivated in TE-polluted soil and unpolluted commercial EO. RI: Retention indices determined in relation to n-alkanes (C-8 to C-40) using ZB-5MS capillary column; RI*: Retention indices obtained from the Pherobase dataset

Area (%)				
**Coumponds**	RI	RI*	**EO (polluted soil)**	**EO (Unpolluted)**
**α-pinene**	929	933	**9.77**	**5.89**
**Camphene**	947	952	0.6	0.24
**Sabinene**	970	973	**26.08**	**8.29**
**β-pinene**	976	980	**3.99**	2.53
**β-Myrcene**	991	991	**5.09**	**4.53**
**o-Cymene**	1025	1020	3.27	-
**D-Limonene**	1030	1031	**5.92**	1.52
**γ-Terpinene**	1059	1059	1.3	0.43
**Trans-sabinene hydrate**	1074	1071	0.23	-
**Linalool**	1105	1107	0.96	-
**α-Thujone**	1110	1111	-	0.43
**Cis-2.8-menthadienol**	1113	1128	0.54	-
**Cis-limonene oxide**	1134	1138	0.36	-
**isopulegol**	1145	1145	0.57	-
**Cis-Verbenol**	1149	1150	1.8	-
**Pinocarvone**	1167	1168	0.5	-
**Methyl phenylacetate**	1176	1176	0.18	-
**Terpinen-4-ol**	1187	1179	0.42	0.31
**Myrtenol**	1200	1202	0.81	-
**Verbenone**	1214	1218	0.17	-
**Bornyl acetate**	1287	1285	0.46	-
**Geranyl acetate**	1382	1383	0.41	2.39
**α-copaene**	1389	1391	-	0.43
**E-beta-damascenone**	1392	1393	0.9	-
**longifolene**	1416	1416	0.26	0.79
**β-Caryophyllene**	1426	1428	1.18	**7.33**
**α-trans-bergamotene**	1436	1434	0.47	1.09
**β-Calarene**	1445	1445	-	0.46
**aromadendrene**	1456	1456	-	1.48
**E-β-farnesene**	1457	1458	1.1	-
**alloaromadendrene**	1461	1461	-	2.88
**γ-Curcumene**	1479	1479	-	0.63
**α-curcumene**	1487	1487	-	2.61
**(+)-β-selinene**	1496	1492	-	**4.65**
**cuparene**	1502	1502	-	1.98
**β-Bisabolene**	1511	1509	0.53	2.36
**β-cadinene**	1520	1519	-	0.51
**δ -cadinene**	1530	1530	-	0.57
**E-α-bisabolene**	1544	1449	-	1.1
**β-thujaplicine**	1559	1559	0.32	0.41
**germacrene D-4-ol**	1573	1574	-	0.27
**Caryophyllene oxide**	1590	1581	**3.35**	1.93
**Carotol**	1610	1600	**27.53**	**38.23**
**Daucol**	1650	1638	0.18	0.3
**14-hydroxy-caryophyllene**	1667	1667	-	0.27
**Tetradecanol**	1681	1680	0.25	-
**Hydrocarbon monoterpene**			**56.01**	23.43
**Oxygenated monoterpene**			7.32	3.13
**Hydrocarbon sesquiterpene**			4.32	**28.06**
**Oxygenated sesquiterpene**			**31.06**	**41.32**
**Others**			0.81	0.9
**Total identified**			**99.52**	**96.84**

The variations in compound proportions and molecular classes between the two EO profiles can be attributed to multiple factors. While the extraction method has a substantial influence on the chemical composition of EO (Messaoudi et al. 2021; Sefidkon et al. 2006), the environmental conditions (specifically soil contamination) appear to be a driving factor in shaping the chemical profile observed in this study (Hubai and Kováts 2024).

Our results indicate that TE pollution triggered a shift in secondary metabolism, favoring the biosynthesis of monoterpenes. Indeed, compounds such as sabinene and D-limonene showed significantly higher levels in the EO of carrots cultivated in TE-polluted soils compared to the commercial oil. This aligns with findings on *Mentha pulegium* L., where treatments with Cu and Zn significantly increased the concentrations of monoterpenes like sabinene, α-pinene, and thymol (Lajayer et al. 2017). Similarly, elevated Pb levels significantly enhanced EO yield and modified the chemical composition of *Mentha crispa* L., notably increasing carvone content (Sá et al. 2015). Although some studies on mint species report a reduction in monoterpenes under TE stress (Prasad et al. 2010), our findings suggest that *Daucus carota* employs a tolerance mechanism involving the upregulation of specific terpenes. This specific accumulation of monoterpenes can be explained by their crucial role in alleviating oxidative stress. These metabolites, including terpenes and monoterpenes (e.g., limonene, linalool), are capable of neutralizing reactive oxygen species (ROS), thereby protecting plant cells from oxidative damage (Bartwal et al. 2013; Figueiredo et al. 2008; Hall 2002; Hossain et al. 2012; Yadav 2010). Therefore, the high concentrations of sabinene and D-limonene observed in our study likely reflect an adaptive response to TE-induced stress. Furthermore, previous studies indicate that plants under TE stress can alter their volatile organic compounds (VOC) profiles and EO biosynthesis pathways. For example, *Tetradenia riparia* L. under Zn stress produced adaptive responses like methanol emission (Bibbiani et al. 2018), while *Mentha piperita* L. under Cd stress showed changes in gene expression related to terpene synthesis, highlighting the impact of TE on EO metabolism (Azimychetabi et al. 2021).

### Essential oil quality

The quantification of TE, including Cd, Pb, Zn, Cu, Cr, and Ni, in EO extracted from carrots grown in polluted soils was conducted and compared to the commercial EO. As shown in Table 5, all measured TE concentrations in both EO were below the limit of quantification (LOQ). This aligns with findings from previous studies; for instance, coriander and sage cultivated in similarly polluted soils also yielded EO with TE levels below the LOQ (Raveau et al., 2021a). Likewise, research on *Anethum graveolens*, *Mentha piperita*, and *Ocimum basilicum* reported undetectable levels of Cd, Pb, and Cu in their EO, despite accumulation in plant tissues (Zheljazkov et al. 2006). Additionally, although *Cymbopogon citratus* grown in red mud-polluted soils accumulated several TE in its biomass, no detectable levels were found in its EO (Gautam and Agrawal 2017). The extraction process of EO from carrot seeds produces a considerable amount of post-extraction residues (Table 5). These residues contain TE concentrations that are significantly lower than those found in the seeds themselves.

**Table 5:** Concentrations of TE (μg/g dry matter (DM)) in EO extracted from carrots grown in TE-polluted and non-polluted soil (commercial EO) and in residues remaining after the distillation process. Data are expressed as mean ± SD

Metals (μg/g DM)	Carrot		
	**TE-polluted soil**		**Non-polluted soil**
	**Post-extraction residues**	**Essential oil**	**Commercial essential oil**
**Cd**	**3.58 ± 0.07**	** <0.1**	** <0.05**
**Pb**	** < 0.5**	** <0.5**	** <0.1**
**Zn**	**55.92 ± 0.41**	** <0.1**	** <0.05**
**Cr**	**1.93 ± 0.10**	** <0.05**	** <0.05**
**Cu**	**13.23 ± 0.15**	** <0.05**	** <0.05**
**Ni**	** < 0.1**	** <0.1**	** <0.05**

When compared with the French regulatory limits for organic amendments (NF U 44–051:2019), all metals exceed the permitted thresholds except Pb, which remains below the LOQ.

These concentrations are similar to those reported for coriander (also used for similar). 

Purposes) whereas sage displays values below the limits set by both the French standard NF U 44–051 (2019) and the European Regulation (EU) 2019/1009 on fertilizing products (Perlein et al. 2021). This indicates that the use of distillation residues from carrot seeds obtained after hydrodistillation, which contain TE (particularly Cd and Zn) for composting or as soil amendments is limited and would require further investigation before being deemed feasible.

### Antifungal and antigerminative activities of carrot seed essential oil

The antifungal activity of carrot seed EO (Table 6) was evaluated in vitro by direct contact against *Fusarium culmorum* and *Zymoseptoria tritici*, two major phytopathogens responsible for root rot and fusarium head blight in cereals, and septoria in wheat, respectively. The EO showed the highest efficacy against *F. culmorum* (IC50 = 0.57 mg/mL), followed by *Z. tritici* (IC50 = 1.09 mg/mL). In comparison, the positive control (Aviator XPro) exhibited significantly lower IC50 values, with 0.23 mg/mL for *F. culmorum* and 0.08 mg/mL for *Z. tritici*. The antigerminative activity of the EO was also tested against *Blumeria graminis*, the causal agent of cereal powdery mildew. In contrast, the positive control (Aviator XPro) demonstrated a significantly lower IC50 value of 0.33 mg/mL for *B. graminis*.

**Table 6:** The antifungal activity of carrot seed EO evaluated using IC50 (mg/ml) against *Fusarium culmorum* and *Zymoseptoria tritici*, and its antigerminative effect on *Blumeria graminis*. Results are presented as mean ± SD, with statistical significance (p < 0.05) determined by the SNK test

	Antifungal activity		Antigerminative activity
	*Fusarium culmorum*	*Zymoseptoria tritici*	*Blumeria graminis*
IC50 EO (mg/ml)	0.57 ± 0.11a	1.09 ± 0.12b	1.47 ± 0.15c
IC50 Aviator (mg/ml)	0.23 ± 0.09b	0.08 ± 0.07a	0.33 ± 0.05c

The EO extracted from carrot seeds in this study showed significant antifungal activity against *F. culmorum* (S4) and *Z. tritici*, as well as notable antigerminative effects against *B. graminis* *in vitro* (S5). Due to its diverse chemical composition, previous studies have reported that carrot seeds’ EO shows antifungal properties against a range of fungi, including *Aspergillus*, *Candida* species, dermatophytes, and *Cryptococcus neoformans* (Maxia et al. 2009; Tavares et al. 2008; Valente et al. 2015). In the present study, the EO obtained from carrot seeds cultivated in TE-polluted soil was rich in oxygenated sesquiterpenes and hydrocarbon monoterpenes. The main constituents identified were carotol, β-caryophyllene, sabinene, α-pinene, and D-limonene. Notably, carotol, daucol, and β-caryophyllene were isolated and shown to inhibit *Alternaria alternata* (Jasicka-Misiak et al. 2004). Purified carotol alone demonstrated strong antifungal activity against several plant pathogens, completely suppressing spore germination of *B. sorokiniana* (Kataria et al. 2017). Sabinene, another major constituent, also exhibits antifungal activity against multiple species, including saprophytic fungi like *Penicillium notatum*, as well as *Ceratocystis polonica* and *Ophiostoma penicillatum* (Judžentienė et al. 2024). However, major compounds are not always the primary contributors of fungal inhibition. Minor constituents, such as α-pinene, β-pinene, β-myrcene, and D-limonene, may play a key role, as observed in carrot seed EO and other plant EOs, including *Citrus sinensis*, which show significant antifungal effects against *Aspergillus flavus* (Ammad et al. 2024; Viljoen et al. 2003; Shi et al. 2019; Velázquez-Nuñez et al. 2013). Minor constituents may also act synergistically with major compounds, enhancing overall antifungal activity (Bhattacharya et al. 2024; Tserennadmid et al. 2011).

Carrot EO exerts its antifungal effect primarily by targeting and destabilizing the fungal cell membrane, which is critical for controlling nutrient exchange and stress response regulation (Peng and Chen, 2024). Ergosterol, a key membrane sterol, which ensures proper cell function, development, and structural stability (Kowalczyk et al. 2020), may have its biosynthesis disrupted by carrot seed EO through multi-step pathway interference and downregulation of key genes such as *ERG11*, *ERG6*, and *ERG4*, mirroring mechanisms observed for cinnamaldehyde in *Fusarium sambucinum* (Wei et al. 2020).

Indeed, EO from various plants have been shown to disrupt ergosterol synthesis, increase membrane fluidity and ionic permeability, inhibit β-glucan and chitin synthesis, damage cell walls, disrupt cellular homeostasis, and induce fungal cell death (Akachoud et al. 2022; Lagrouh et al. 2017).

### Herbicidal and antigerminative effects of carrot seed essential oil (*in vitro* assays)

Herbicidal and antigerminative effects of carrot seed EO in vitro are presented in Table 7, showing IC50 values for two types of activity (herbicidal and antigerminative) on *Lolium perenne* (ryegrass) and *Lactuca sativa* (lettuce), and comparing carrot seed EO from plants grown in TE-polluted soil to glyphosate, a reference herbicide. For herbicidal activity, evaluated through root length measurements with EO concentrations ranging from 0.004% to 1%, the EO showed notable efficacy on *L. perenne* with an IC50 of 0.11 mg/mL, while glyphosate was slightly more effective at 0.07 mg/mL. In contrast, on *L. sativa*, the EO was less effective (IC50 = 0.58 mg/mL) compared to glyphosate (IC50 = 0.02 mg/mL). These results indicate that the efficacy of carrot seed EO varies with plant species, showing stronger herbicidal activity against *L. perenne* than *L. sativa*. Regarding seed germination, carrot seed EO and glyphosate showed similar activity on *L. perenne*, with IC50 values of 0.17 mg/mL and 0.14 mg/mL, respectively.

**Table 7:** IC50 (mg/ml) for the herbicidal and antigerminative activity of carrot seed EO evaluated on two plants, ryegrass and lettuce. Data are expressed as mean ± SD. Values not sharing a common letter indicate a significant difference (p < 0.05) based on the t-test

	Herbicidal activity		Antigerminative activity	
	*Lolium perenne*	*Lactuca sativa*	*Lolium perenne*	*Lactuca sativa*
IC50 EO (mg/mL)	0.11 ± 0.01a	0.58 ± 0.91b	0.17 ± 0.11 a	0.51 ± 0.02 b
IC50Glyphosate (mg/mL)	0.07 ± 0.01a	0.02 ± 0.01 a	0.14 ± 0.08 a	0.15 ± 0.06 a

Previous studies have shown that EO can exert phytotoxic effects on a wide range of plant species, affecting both crops and weeds (Amri et al. 2023; Jouini et al. 2020; Pérez-Izquierdo et al. 2022). The inhibitory effects of EO on seed germination are generally attributed to allelopathic compounds, which can disrupt cell membranes or interfere with DNA and RNA synthesis (Abd El-Gawad 2016; Benchaa et al. 2019).

In the present study, carrot seeds’ EO exhibited significant inhibitory effects on both seed germination and root growth of the tested plant species. Complete inhibition of germination was observed for *Lolium rigidum,* a monocotyledon, at the highest concentrations (1% and 0.5%) (S5), and for *Lactuca sativa*, a dicotyledon, at 1%. These results are consistent with previous studies reporting that Mediterranean aromatic plant EO exerts dose-dependent phytotoxic effects on *Raphanus sativus*, while *Cistus ladanifer* EO strongly inhibits tomato germination (De Almeida et al. 2010). The EO of carrot seeds grown in polluted soils contained high levels of monoterpene hydrocarbons (56.01%), which are known for their herbicidal properties (Amri et al. 2023; Chowhan et al. 2011). Similarly, the EO of *J. horizontalis* contain significant proportions of monoterpene hydrocarbons, confirming their phytotoxic effect on lettuce (Mirmostafaee et al. 2020). Sabinene, present in significant amounts in carrot seed EO, has also been reported to inhibit shoot and root growth in both monocots (*Poa annua*) and dicots (*Amaranthus retroflexus*) when present in *Dracocephalum integrifolium* EO (Zhou et al. 2019). Other compounds, including α-pinene, β-pinene, β-myrcene, p-cymene, and γ-terpinene, identified in the EO of *D. tortuosa,* have also been shown to exhibit phytotoxic and herbicidal activities (Chowhan et al. 2011; Martino et al. 2010). Application of α-pinene and β-pinene to seeds of *Cassia occidentalis* and *Oryza sativa* reduced chlorophyll synthesis and decreased the activity of hydrolytic enzymes such as proteases, α-amylases, and β-amylases, while increasing the activity of peroxidase, catalase, superoxide dismutase, ascorbate reductase, and polyphenol oxidase, indicating oxidative stress induced by these compounds (Chowhan et al. 2011; Singh et al. 2006). Furthermore, pinenes can disrupt energy metabolism in maize seedling roots by inhibiting oxidative phosphorylation and interfering with the electron transport chain (Zengin and Baysal 2014). Overall, carrot seed EO exhibits both herbicidal and antigerminative effects on monocots and dicots, with stronger activity at higher concentrations and particularly on monocotyledons, highlighting its potential as a natural weed control agent.

## Conclusions

The present study highlights the promising potential of carrot cultivation in TE-polluted soils as a phytomanagement strategy, combining effective phytoextraction, EO production, and enhancement of soil microbial activity. The carrot *Daucus carota* L. demonstrated a remarkable capacity to extract TE, particularly Zn, with a high translocation factor (TF of 4.38), indicating efficient translocation from roots to shoots. Significant Cd uptake was also observed, evidenced by a BCF of 1.71, which confirms the species' high potential for Cd accumulation in plant tissues. Beyond metal uptake, carrot cultivation improved soil quality by stimulating microbial biomass, a key indicator of soil fertility and biological activity. The aerial biomass can be efficiently valorized via hydrodistillation to produce high-quality EO. These EO were free from detectable levels of TE, making them appropriate for non-food applications .

These EO also exhibited notable antifungal activity against *F. culmorum*, *Z. tritici*, and *B. graminis*, as well as herbicidal and antigerminative effects on ryegrass and lettuce, highlighting their potential for agricultural use in plant protection. However, the residual biomass contained high levels of TEs, limiting its further valorization. Overall, these results highlight the combined ecological and economic potential of *Daucus carota* L. as a phytomanagement crop in TE-contaminated soils. Future research should focus on optimizing cultivation techniques, exploring additional valorization pathways for contaminated biomass, and evaluating the long-term effects of this approach on soil health and crop productivity.

## Supplementary Information

Below is the link to the electronic supplementary material.

ESM1 (DOCX 3.98 MB)

## Data Availability

The datasets generated and analyzed during the current study are available from the corresponding author on reasonable request.
