# Supplementary material for: Essential oil production from seeds of carrot (Daucus carota L.) grown on phytomanaged trace element-contaminated soils
Source: Environ Sci Pollut Res Int. 2026 Feb 7;33(8):3189–205. doi: 10.1007/s11356-026-37466-9 (PMC13005824; doi:10.1007/s11356-026-37466-9)
Supplement: Supplementary file 1 — (DOCX 3.98 MB) [file 11356_2026_37466_MOESM1_ESM.docx]

**Supplementary materials**


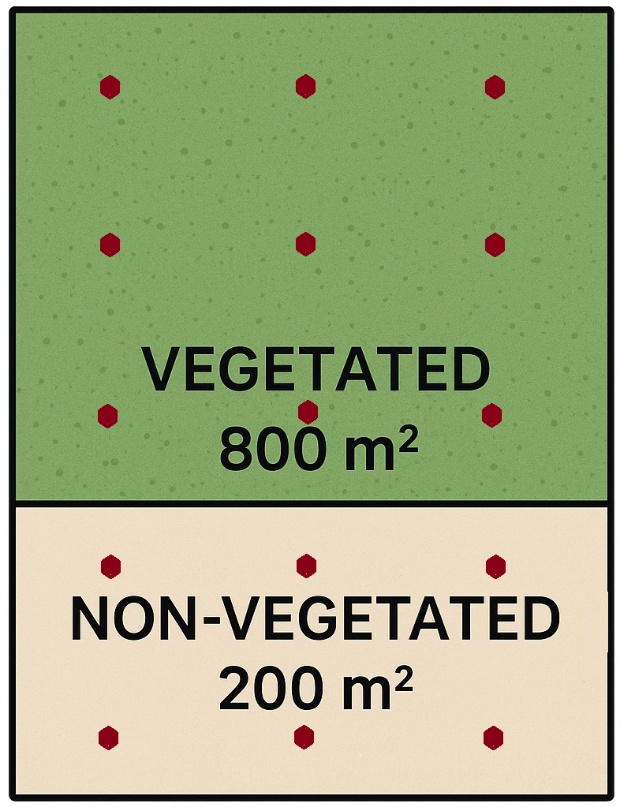


**S1. Experimental design showing the 800 m^2^ vegetated plot and the adjacent 200 m² unvegetated area. The red points indicate the geo-referenced sampling locations: nine in the vegetated plot and six in the unvegetated plot, evenly distributed to best represent the configuration of the plots.**


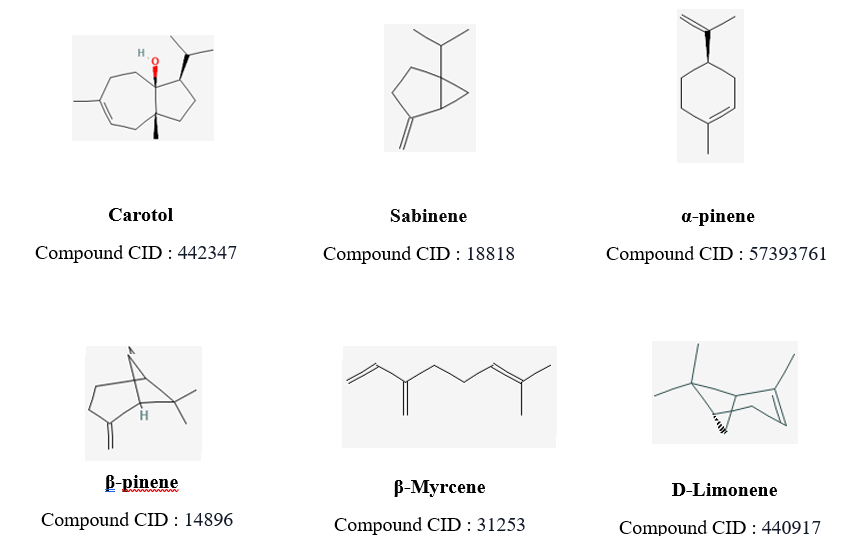


**S2: The structure of the major chemical compounds found in the oils of carrot seeds grown in polluted soils (according to the PubChem database).**


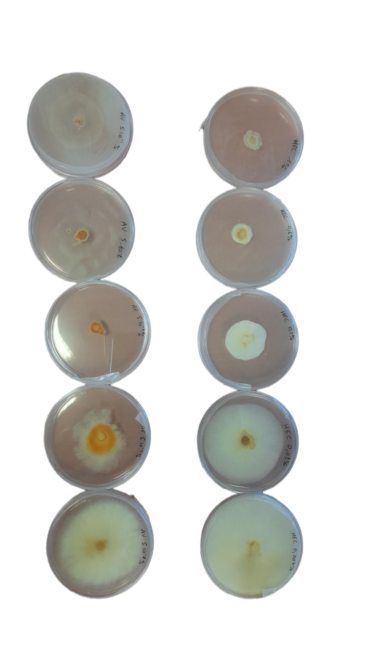


B

A

1%

0.5%

0.1%

0.02%

0.004%

0.5%

5×10⁻^2^ %

5 ×10⁻^3^ %

5 ×10⁻^4^ %

5 ×10⁻^5^ %

**S3: The antifungal activity of EO extracted from carrot seeds was assessed in vitro against F. culmorum. Two treatments were evaluated: (A) EO at concentrations ranging from 0.004% to 1%. and (B) a commercial fungicide (aviator).**


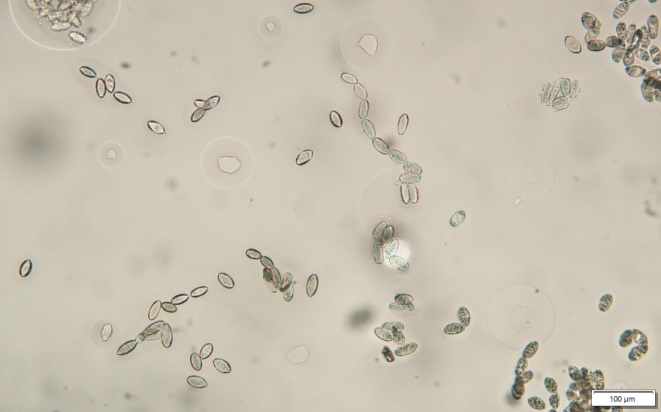

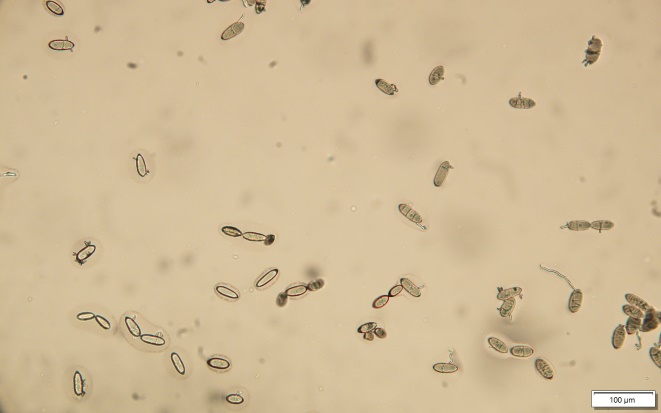


A

B

**S4*:* The antigerminative activity of EO extracted from carrot seeds was evaluated in vitro against B. graminis. Two conditions were compared: (A) a negative control consisting of agar/DMSO (germinated spores) and (B) EO at a concentration of 1%. The results were visualized and analyzed under a microscope.**


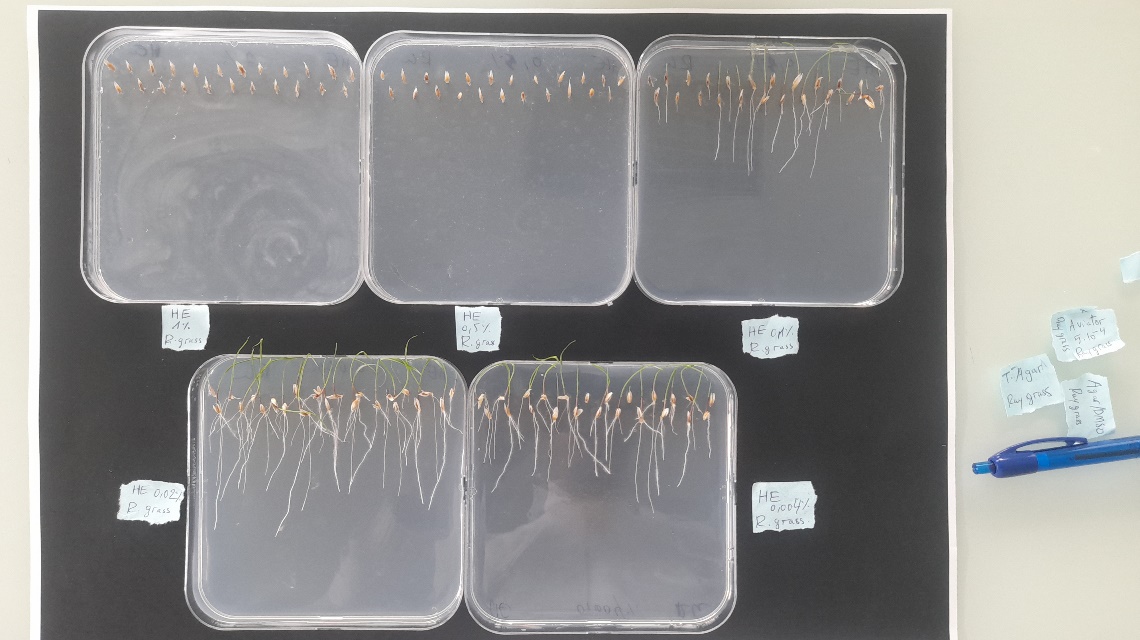

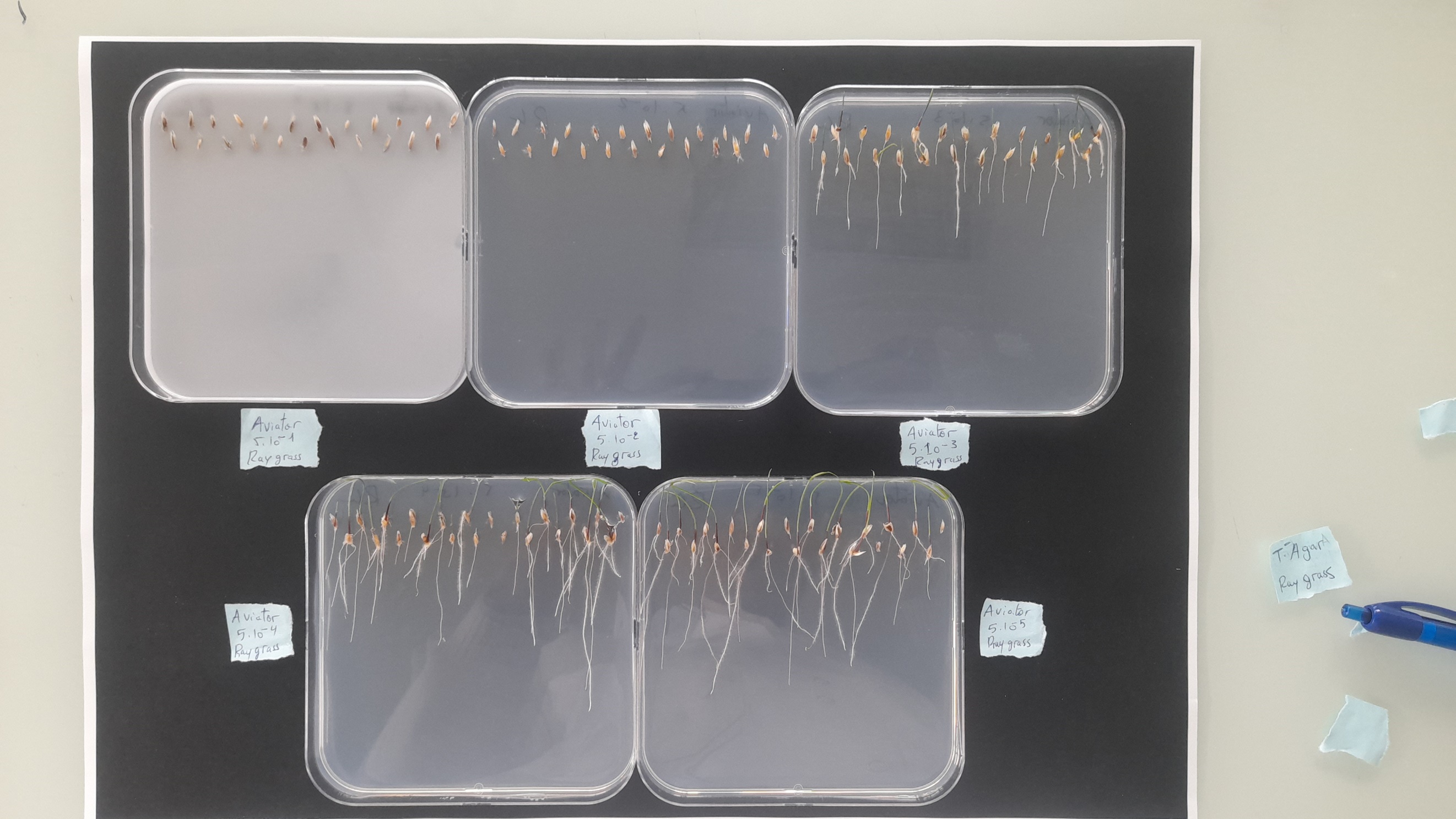


A

B

1%

0.5%

0.1%

0.02%

0.004%

5×10⁻^2^ %

5 ×10⁻^3^ %

5 ×10⁻^4^ %

5 ×10⁻^5^ %

**S5: Antigerminative and herbicidal effects of EO extracted from carrot seeds on rye grass. Panel A shows the results of EO tested at five concentrations ranging from 0.004% to 1%. while Panel B presents the effects of glyphosate for comparison.**

**Highlights**

• Carrot efficiently extracts Cd and Zn from trace element-contaminated soils.

• Cultivation promotes soil microbial biomass and community structure shifts.

• Carrot seed essential oils are free of detectable toxic trace elements.

• Oils are mainly composed of carotol (27.5%) and sabinene (26%).

• Oils exhibit strong antifungal, antigermination, and herbicidal activities.
